# Cocaine and Its Salts

**Published:** 1885-02

**Authors:** E. Merck

**Affiliations:** Darmstadt


### Translations.

---

COCAINE AND ITS SALTS. *Translated by* WM. M. SMITH, M.D.

$C_{17} H_{24} NO_4$ (Lossen).

Cocaine is an alkaloid of the coca leaves (Erythroxylon Coca Sam.), which was isolated by Niemann in 1860. In 1862 Lossen found a second, volatile, base, hygrin, which has been little investigated up to the present time; but it is apparently weak, and without the characteristic action of the cocaine. Other extracts of the leaves are: Ecgonin, coca tannic acid, and a peculiar wax. The cocaine crystals belong to the monoclinic system; they melt at 98° C., dissolve easily in alcohol, still more easily in ether, but only in 704 parts water at 12° C. The salts of cocaine, on the contrary, are readily soluble in water.

The first intelligence of the action of coca, taken internally, was furnished in the 16th century (Dr. Monardes, Sevilla, 1569). In 1749, the plant was brought to Europe, described by Jussie, and named *Erythroxylon coca*, by Lamarck. Tschudi, Markham, Poppig, and other investigators who traveled in South America, observed that the natives chewed the coca leaves when they wished to neutralize the effects of over-fatigue. The Indians macerate the leaves with ash of chenopodium quinoa, in order to eliminate the tannic acid by means of the alkali, and to free the alkaloid.

I produce the cocaine-alkaloid pure, as also its combinations, with muriatic acid, salicylic acid, hydrobromic acid, tartaric acid, and citric acid. Since the production of the cocaine has been accomplished, it is believed that in this substance the active principle of the coca leaves has been found.

At first it seemed probable that a similar action might be found as a quality of one of the analogous alkaloids, such as caffein, thein, or theobromin. However, nothing has yet occurred to support this idea.

Cocaine acts upon the nerve centers, but also upon other nerve regions—in small doses, as a stimulant; in large ones it causes paralysis. It kills warm-blooded animals, by arresting pulmonary action, though they are less affected by it than the cold-blooded animals. Although no doubt exists, therefore, that cocaine is a poison, still its toxic qualities are relatively small, and its action is not cumulative.

Schroff, who in 1862 made the first experiments with the remedy, saw, in puppies, after a dose of 0.05 grm. *per os.*, fluctuating respiration and transitory mydriasis. The same dose, administered subcutaneously, caused the death of the animal experimented upon, with epileptiform convulsions and very decided mydriasis, which immediately disappeared as soon as death occurred. With frogs, the application of 0.001 grm. is followed by complete loss of the power to move; *dosis lethalis* 0.002 grm.

According to Fronmüller, who, in 1863, examined the narcotic action of cocaine, 0.03 to 0.33 grm. administered to man internally produced no important effect; in one case, sleep intervened. Pulse and breathing were somewhat accelerated at first, later were subnormal. On one occasion, when taken with suicidal intent, 1.5 grm. cocaine was not followed by any serious effect upon the health. The fatal dose for man, therefore, must be very great if it cannot be established that the preparations of that date were not pure cocaine.

So far as the coca infusions have been experimented with, it may be considered that the leaves contain between 0.02 to 0.2 per cent. of cocaine. Of my cocaine mur. sol., 0.05 grm. appears to be an effective dose for man.

After subcutaneous injection of an attenuated solution of cocaine in man, a sensation of warmth is felt at first, then loss of sensation in the region about the injection; finally, a circumscribed redness of the skin, and after about thirty minutes, a return to the normal condition. Applied to the tongue, it benumbs the nerve sensibility of the same.

Quite lately, Dr. Th. Aschenbrandt, in the No. 50 (1883) of the *Deutsche Med. Wochenschrift*, has appeared as a champion of the cocaine, in that he accredits it with most remarkably beneficial qualities in great debility, particularly that produced by diarrhœa. During the last month, Prof. Dr. E. v. Fleischl, in Vienna, and Dr. Sigm. Freud, Physician in the General Hospital in Vienna, have diligently occupied themselves with this preparation. The former, particularly, has determined that the cocaine, by hypodermic injection, has proved itself to be an invaluable adjuvant against the continued use of morphia; also, against a single fatal dose. This fact alone should give the remedy an enduring place among the treasures of the physician.

Those above referred to have given the medicine in the form of its muriatic acid combination, in doses of 0.05 to 0.15 grm., and as much as 0.5 grm., in a watery solution, has been given per day. Dr. Freud has made a number of experiments upon himself and others, and besides a constant increase in physical strength, he has recognized a true coca-euphrasy. The feeling of hunger and the want of sleep disappear during the action of the coca.

With the attempt, in the following lines, to answer the question as to the therapeutic worth of the cocaine, I must likewise remark that up to the present time only the foundation can be given for future research. To this end, the remedy will be diligently experimented with in the various fields of medical science, so that it may be hoped that decided results as to its real worth may be obtained at an early date.

Cocaine is a stimulant which is peculiarly adapted to elevate the working ability of the body, without any dangerous result. Its action is stronger than that of alcohol. Its use for this purpose in marching or mountain climbing is self-evident. The dose in such cases may be from 0.05 to 0.01 grm., repeated as required.

It is still an open question as to whether mental labor may be carried on a greater length of time or made lighter by its use or not. Even so must it remain undetermined for the present as to whether the psychiater will be able to make use of cocaine for the purpose of inducing a continued elevation of the powers of the nerve centers. The subcutaneous use of cocaine in a daily dose of from 0.0025 to 0.1 grm. has been applied for months to patients suffering from melancholy, with some definite results.

Cocaine is a stomach remedy, in so far that after debauches in eating and drinking, it has produced rapid amelioration, and a normal longing for food, when used in doses of from 0.025 to 0.05 grm.

In atonic weakness of digestion and nervous disturbances of the stomach, a lasting return to the normal condition may be attained from time to time by the use of cocaine.

Also in cachexia is the continued use of cocaine recommended: in phthisis, great anæmia and wasting fevers. Further threatened mercurial cachexia from the continued use of quicksilver has been avoided by the incorporation of cocaine.

In any event, cocaine has its greatest future in morphia, and perhaps, also, in alcohol-abstinence. An American, W. H. Bentley, published in 1878 the observation that coca may paralyze the morphium hunger of the opium-eater. If all that has been recently published in this connection should be confirmed, the remedy is of incalculable worth. Relapses do not occur; on the contrary, the disuse of the coca may take place promptly at the proper time without a return of the morphium hunger. Depression and nausea do not occur during the cure; diarrhœa and chills are the only symptoms observable.

In case of gradual or long-continued withdrawal of opium, decreasing doses of morphia and increasing doses of cocaine are given. In cases of absolute and sudden abstinence, doses of 0.1 grm. are injected as often as the morphium hunger is felt. Confinement in institutions become quite unnecessary with this method. Dr. Freud, who, with others, saw such a case, after ten days cocaine treatment (0.1 grm. subcutaneously three times per day), pass into positive convalescence, is of the opinion that a direct antagonism exists between morphium and cocaine.

The treatment for the alcohol habit is far more difficult. The first experiments date also from America, and seem to have turned out favorably.

The remedy has also been recommended as an aphrodisiac, and Dr. Frend has undoubtedly observed sexual excitation occur after the use of cocaine.

As already remarked, as soon as cocaine comes in contact with the mucous membrane it produces a transitory loss of sensation of the same. Therefore not only attempts to cure certain laryngeal and throat affections are made, but it is hoped that in operations in the larynx it may be employed as a local anæsthetic. An important and obviously frequent employment of cocaine seems to be assured in the field of ophthalmology.

On the 15th of September, at the meeting of the Ophthalmological Society in Heidelberg, the experiments of Dr. Koller, instituted in Vienna, were discussed. Dr. Koller has experimented on the eyes of animals, also upon his own, a number of times, and has found that immediately after dropping in a 2 per cent. solution of cocaine mur., a brief burning occurs, continued for not more than a half minute, which is soon succeeded by an uncertain feeling of dryness. The opening of the lids of the experimented eye appears wider; reflex action, which otherwise occurs upon approaching the cornea, motion of the head, of the lids, and shrinking back of the eyeball, disappears. In this condition a small scoop can be passed over the cornea without producing an unpleasant sensation; or the conjunctiva bulbi may be taken up with the forceps.

The anæsthesia of the eye lasts about ten minutes, though lack of sensation may persist some hours. Twenty to thirty minutes after the instillation the pupil dilates and returns to its normal state in a few (say twelve) hours. A slight, easily-overcome paralysis of the accommodation during this time is the only abnormality observed. As to the rest, the functions of the eye remain intact.

Dr. Koller has determined the anæsthetic action of the cocaine in animals in which he had produced a keratitis by irritation with a foreign body. He prognosticates a future for the cocaine in the removal of foreign bodies from the cornea, and in greater operations (cataract-extractions, *iridectomy*), or as a narcotic in corneal and conjunctival affections. Which salt of cocaine can be used to the greatest advantage in eye-practice will be very shortly determined.

It remains to be said that the experiments, the results of which I have here reported, have been made, without exception, with the preparations brought into commerce under the name of "*Cocain mur. solut. Merck;*" only for these are the doses and action, as above stated, to be relied upon.

E. MERCK.

Darmstadt, October, 1884.—*Klinische Monatssblätter für Augenheilkunde, Zcherder.*

